# Glomus tumor of the shoulder: A case report and review of the literature

**DOI:** 10.3892/ol.2013.1478

**Published:** 2013-07-19

**Authors:** AGNESE PROIETTI, GRETA ALÌ, FRANCESCA QUILICI, PIETRO BERTOGLIO, ALFREDO MUSSI, GABRIELLA FONTANINI

**Affiliations:** 1 Division of Pathological Anatomy, Department of Surgery, Medical, Molecular and Critical Area Pathology, University of Pisa, Pisa, Italy; 2 Division of Thoracic Surgery, Department of Surgery, Medical, Molecular and Critical Area Pathology, University of Pisa, Pisa, Italy

**Keywords:** glomus tumour, glomangioma, shoulder, extradigital glomus tumour, review

## Abstract

Glomus tumors are benign neoplasms that arise from neuromyoarterial glomus bodies, with clinical manifestations that include acute pain, cold intolerance and tenderness. Glomus tumors may occur anywhere in the skin, soft tissue or gastrointestinal tract, but are most frequently encountered in the nail bed of the hands. The present study reports the case of a 30-year-old female with a history of shoulder pain caused by a cystic neoformation. Following surgery, a microscopic examination revealed nests of small cells of a rounded and regular shape. The tumor cells exhibited positive expression for CD34 and smooth muscle actin. This study supports and confirms the fact that a glomus tumor is a benign neoplasm that may occur in multiple locations. Therefore, the significance of a histological and immunohistochemical approach for a correct characterization of this lesion is required.

## Introduction

Glomus tumors are neoplasms that arise from modified smooth muscle cells of the glomus body, which is a specialized form of an arteriovenous anastomosis that plays a significant role in the regulation of skin circulation (1,2). Glomus tumors were first described in 1924 by Masson, who compared the tumors with the normal glomus body and suggested that the lesion represented hyperplasia or overgrowth of this structure (3). It is now well accepted that the lesions are neoplastic.

Glomus tumors have been reported to account for 1–6% of all soft-tissue tumors and 1–5% of hand tumors (4). The tumors may occur during adult life at 20–40 years of age and are equally represented in males and females. In a large series by Beaton and Davis (5), extradigital tumors were more common in males, while subungual lesions predominantly affected females. Classic glomus tumors are typically solitary, though they may rarely occur as multiple nodules (2,6,7). Multiple lesions have been reported in neurofibromatosis-1 (8). Malignant transformation is rarely reported (9–11).

Clinical manifestations include paroxysms of pain, cold sensitivity and point tenderness. In certain patients the pain is accompanied by additional signs of hyperesthesia, muscle atrophy or osteoporosis of the affected area (12).

Grossly, the tumor is a small purple nodule that varies between 2 and 20 mm in diameter, though tumors of >3 cm have been reported (13). Histologically, the tumors have variable quantities of glomus cells, blood vessels and smooth muscle cells. Accordingly, they are classified as solid glomus tumors, glomangiomas and glomangiomyomas (14). Solid glomus tumors are the most common subtype (73%), followed by glomangiomas (25%). Glomangiomyoma is the rarest variant with a frequency of 8% of all glomus tumors (15).

The present study reports a case of a glomus tumor of the shoulder and discusses the histological and immunohistochemical features.

## Case report

### Patient and treatment

A 30-year-old female was referred to the Department of Thoracic Surgery (University of Pisa, Pisa, Italy) with a 1-year history of shoulder pain and paresthesia of the left arm. Upon physical examination, the range of motion of the left shoulder was normal.

**Figure 1:**
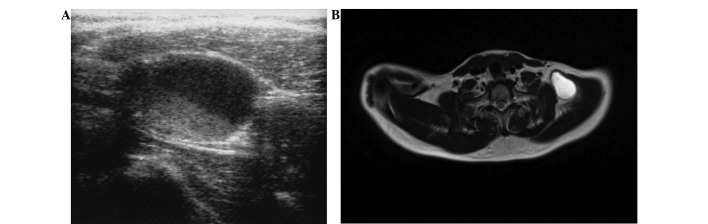
(A) Ultrasound scan showing a well-defined hypoechoic mass. (B) MRI scan showing a hyperintense lesion on the T2-weighted image.

Ultrasonography of the supraclavicular area revealed a well-defined hypoechoic oval mass measuring ~3.5 cm. MRI of the shoulder confirmed the presence of a cystic neoformation measuring ~4 cm located between the trapezius and levator scapulae muscles (Fig. 1).

The patient was treated by a wide excision of the mass with a surgical incision in the neck, laterally to the sternocleidomastoid muscle. The patient did not undergo any further treatment following the excision and did not have any recurrence.

### Specimens

Excised specimens were fixed in 10% neutral buffered formaldehyde and embedded in paraffin. Routine hematoxylin and eosin staining was performed on the microscopic section for histopathological examination.

### Immunohistochemistry

A paraffin block was chosen for immunohistochemical study. An immunohistochemical evaluation was performed using the avidin-biotin-peroxidase complex method. Antibodies were purchased from Ventana Medical Systems (Tucson, AZ, USA). The antibodies that were used were mouse monoclonal anti-smooth muscle actin, mouse monoclonal anti-CD34, rabbit monoclonal anti-CD31, polyclonal anti-factor VIII, mouse monoclonal anti-CK-Pan, mouse monoclonal anti-CD68, mouse monoclonal anti-S-100, mouse monoclonal anti-CD99, polyclonal anti-calretinin and mouse monoclonal anti-desmin. All the antibodies were pre-diluted.

The analysis of the specimens was performed by two pathologists and the differential diagnosis was widely discussed.

### Results

The specimens were obtained from a 30-year-old female who underwent an excision of a subcutaneous mass of the supraclavicular area.

The gross appearance of the specimen was a grayish fragmented cystic lesion, measuring 4 cm at the maximum. The cut sections revealed firm tissue without hemorrhagic or necrotic areas.

A microscopic examination showed multiple nests of typical glomus cells surrounded by fibrous tissue with focal myxoid changes. The glomus cells contained epithelioid elements with moderate amounts of clear to eosinophilic cytoplasm and round nuclei with fine chromatin. These cells were closely associated with small vascular channels and nerves. The cells demonstrated a low proliferative activity (Ki-67 <5%) and a low mitotic rate of <1 mitotic figure (MF)/50 high-power fields (HPFs). The lesion was well-circumscribed and there was no evidence of hemorrhage or necrosis.

**Figure 2:**
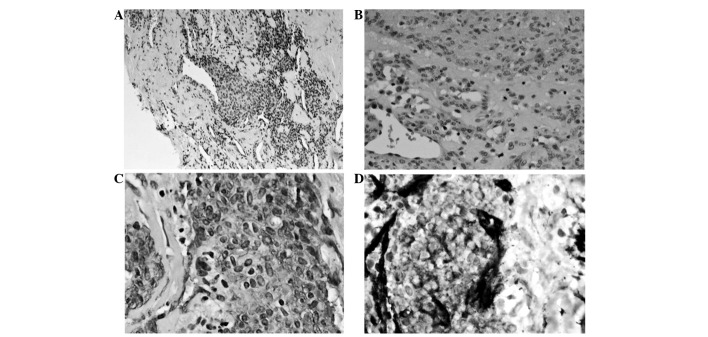
(A) Hematoxylin-eosin stain (magnification, ×10). (B) Hematoxylin-eosin stain (magnification, ×20). (C) Actin immunohistochemistry positive stain (magnification, ×20). (D) CD34 immunohistochemistry positive stain (magnification, ×20).

Immunohistochemical staining was positive for SMA and CD34 and negative for CD31, factor VIII, CK-Pan, CD68, S-100, CD99, calretinin and desmin (Fig. 2).

## Discussion

A glomus tumor is a hamartoma that develops from a neuromyoarterial glomus body and consists of dilated vascular channels surrounded by proliferating glomus and nerve cells. It accounts for 1–6% of all soft tissue tumors and 1–5% of hand tumors (4). Glomic units are located in the stratum reticularis of the dermis throughout the body, but they are highly concentrated in the digits, palms and soles. The units are most frequently encountered in the subungual region, but also in the precoccygeal soft tissue (glomus coccygeum). The glomus body is made of preglomic arterioles derived from the small arterioles that supply the dermis and is lined by plump cuboidal endothelial cells and surrounded by longitudinal and circular muscle fibers. Scattered throughout the muscle fibers are rounded, epithelioid glomus cells (12). The cells are absent in children under the age of 1 year. With advancing age the cells begin to atrophy, while the overall number of glomic units decreases (2).

Glomus tumors are usually located in the deep dermis of the extremities and in the subungual region of the hands. Other sites are the shoulder (16), thigh (17), knee (4) and gastrointestinal tract, including the stomach (18) and liver (19).

Malignant transformation is extremely rare, but possible (10,11). Folpe *et al*(15) proposed the following classification criteria for malignant glomus tumors: i) Deep location and a size of >2 cm; ii) presence of atypical mitotic figures; or iii) combination of moderate to high nuclear grade and mitotic activity (5 MFs/50 HPFs).

The typical presentation of a bluish, painful lesion in either a subungual or digital pulp location is now well recognized by clinicians. However, when the lesion is extradigital, the difficulty in forming a diagnosis often leads to delays and misdiagnosis (20,21).

A review of the literature suggests that the extradigital distribution along the upper extremity may be more frequent than is generally assumed. The forearm has been noted to be the most common extradigital location, while the shoulder and upper back are the least frequent (12).

A glomus tumor of the shoulder has been reported in 14 cases (Table I). According to the cumulative data, including that of the present case, the mean age of the patients was 48.5 years (range, 30–71). There was no gender predominance (six females and six males). The mean size of the tumors was 1.9 cm (range, 0.5–4). Seven tumors (50%) were located in the right shoulder, four (28%) were of the left side and in two cases (14%), the location of the lesion was not reported. No data were available for the cases studied by Beaton and Davis (5) The mean duration of symptoms was 10.75 years (range, 0.5–20 years). The data indicate that in the subcutaneous location of the extradigital areas, the tumor only becomes visible at a late stage, which correlates with the enlargement of the mass. The majority of lesions are only a few millimeters in diameter at the onset of symptoms and this limits the usefulness of palpation in such cases. The absence of objective findings frequently results in a diagnostic delay, a finding that is confirmed by the protracted duration of symptoms observed in the majority of series and case studies. Various diagnostic imaging techniques have been reported to enhance the ability to detect these lesions. The are no specific imaging techniques to aid in the diagnosis. Ultrasonography, despite its low specificity, may aid in locating the lesion. MRI provides more details of the lesion and its association with the adjacent structures (12). It must be emphasized that a diagnosis relies on a high index of clinical suspicion.

The present study reports a case of a glomus tumor in a young patient with a short duration of symptoms in the left arm. The lesion was located in the left shoulder and despite its large size, the patient experienced a short duration of symptoms, including left arm paresthesia. Furthermore, in contrast with data that has been previously reported in the literature, the lesion was cystic and perivascular. A correct clinical diagnosis was obtained from the imaging techniques (ultrasound and MRI). The surgical excision of the lesion resulted in a complete disappearance of the symptoms.

In conclusion, glomus tumors of the shoulder are not as uncommon as previously believed. On this basis, in cases of unexplained pain in this area, a glomus tumor should be considered in the differential diagnosis.

## Figures and Tables

**Table I:** Summary of the shoulder glomus tumor cases.

First author/s, year (ref.)	Age, years	Gender	Side	Size, cm	Duration of symptoms, years
Bailey, 1935 (26)	48	M	L	0.3	20.0
Beaton and Davis, 1941 (5)	NA	NA	NA	NA	NA
Riveros and Pack, 1951 (27)	40	F	NR	0.5	NR
Heys, 1992 (25)	NR	NR	NR	NR	NR
Massey, 1992 (31)	41	F	R	1.0	Several
Yoshikawa, 1996 (29)	35	F	L	4.0	20.0
Roberts, 1999 (23)	67	M	R	3.5	20.0
Ghaly, 1999 (28)	62	M	R	1.0	20.0
Abela, 2000 (16)	52	M	R	1.5	10.0
Solivetti, 2002 (30)	58	M	R	0.4	1.0
Boretto, 2008 (22)	54	F	R	NR	30.0
Gautam, 2008 (32)	25	F	L	NR	5.0
Karakurum, 2009 (24)	71	M	R	2.5	0.5
Present case, 2012	30	F	L	4.0	1.0

M, male; F, female; R, right; L, left; NR, not recorded; NA, not available.
